# Immune Phenotype and Body Condition in Roe Deer: Individuals with High Body Condition Have Different, Not Stronger Immunity

**DOI:** 10.1371/journal.pone.0045576

**Published:** 2012-09-19

**Authors:** Emmanuelle Gilot-Fromont, Maël Jégo, Christophe Bonenfant, Philippe Gibert, Benoit Rannou, François Klein, Jean-Michel Gaillard

**Affiliations:** 1 Université de Lyon, Université Lyon 1, UMR CNRS 5558, Villeurbanne, France; 2 Université de Lyon, VetAgro Sup, Marcy l’Etoile, France; 3 Office National de la Chasse et de la Faune Sauvage, Studies and Research Department, Paris, France; Auburn University, United States of America

## Abstract

An efficient immunity is necessary for host survival, but entails energetic costs. When energy is limited, immunocompetence and body condition should co-vary positively among individuals and, depending on body condition, individuals should allocate more either in innate immunity or in adaptive response. We tested whether immune phenotype depends on body condition in large mammals, using data from two contrasted populations of roe deer *Capreolus capreolus* in France. Roe deer living at Chizé, a forest with poor habitat quality, were expected to show lower values for body condition and immune parameters than roe deer at Trois Fontaines, a forest with high habitat quality. From 285 blood samples collected between December 2009 and March 2011, we measured seven metabolic parameters and ten immunological parameters. A Principal Component Analysis showed that all indicators of body condition co-varied positively and were lowest at Chizé. Several immunological indicators correlated to body condition and differed between Trois Fontaines and Chizé. However, high body condition was not associated to a high average level of immunocompetence, but instead to high levels of indicators of acute inflammatory innate response, while low body condition was associated to high levels of monocytes and lymphocytes, possibly reflecting adaptive immunity. Limited data suggest that the difference between populations was not related to the presence of specific parasite species, however parasite exposure and stress have to be investigated to gain a more complete understanding of the determinants of immunity.

## Introduction

The immune function confers protection to hosts against pathogens, thus identifying the factors causing variation in immunity is required to understand the cost of parasitism in hosts. The immune system first develops following antigen stimulation [1]. However, immune functions are energetically costly to develop, maintain and use [2] thus the energetic balance of individuals should also be considered. When resources are limited, trade-offs should occur between immunocompetence and competing functions. Variability in resource acquisition should thus lead to variable general level of immunocompetence among individuals [3]. Food restriction has been experimentally shown to limit several components of immunity like cellular response against gastrointestinal parasites [4] or levels of antibody [5], [6]. Individual follow-up has also suggested that low body condition is frequently followed by infection in field voles [7].

However, reducing immunological variation to the immunosuppression/enhancement gradient does not fully capture the observed variation in immunity [8]. The immune response includes innate and adaptive components, which interact together but may show different costs and benefits depending on the species and individual considered. The innate response provides a rapid and largely non-specific response, while adaptive immunity confers long-term, more specific protection. Recent experimental work showed that innate immunity, which relies on strong cellular response, is costly to maintain and to use, and entails strong immunopathological costs [9]. In contrast, the cost of adaptive response is mainly due to its development [9]. On the other hand, the benefit of each component differs: in particular, adaptive immunological memory is most useful in individuals that are expected to encounter the same pathogen several times during their life. Building on these observations, Lee [10] proposed the general prediction that, in individuals experiencing the highest energetic demands, the costliest defences, such as the innate inflammatory response, should be down-regulated, while the cheapest defenses (such as adaptive antibody-based response) should be up-regulated. This prediction should apply at both interspecific, with fast-living species expected to have more innate-oriented immunity than slow-living species, and intra-specific levels, with individuals experiencing strong energetic demand predicted to have relatively high levels of adaptive immunity. In mice and chicken, comparing lineages selected for fast-living and slow-living traits confirmed these predictions [11], [12]. In mallards, innate and adaptive immunity responded differently to a fasting-refeeding experiment [6]. Whether the innate/adaptive balance correlates with body condition (a proxy of an individual’s ability to acquire resources) in natural populations has received little empirical support despite body condition correlated to innate response in several bird species [13], [14], [15].

The roe deer *Capreolus capreolus* is a relevant model to test the relationship between immune phenotype and body condition in the wild: having low body reserves [16], roe deer cannot compensate for high immunological effort. We measured body condition and immunological parameters in roe deer captured in two populations with contrasting food resource availability. We expected that (H1) body condition should be higher in the population having access to more food resources, (H2) overall, immune parameters should increase with body condition, and (H3) immunity should be oriented towards innate response when body condition is high.

## Materials and Methods

### Ethics

All necessary permits were obtained for the described field studies. The protocol of capture and blood sampling of roe deer under the authority of the ONCFS have been approved by the Director of Food, Agriculture and Forest (Prefectoral order 2009–14 from Paris). This permission is given at the national level. The land manager of both sites, the Office National des Forêts (ONF), permitted the study of the populations (Partnership Convention ONCFS-ONF dated 2005–12–23).

### Study Sites and Captures

The Chizé forest is located in Western France (46°05′N, 0°25′W). With a low productivity, this forest offers a poor habitat to roe deer. In contrast, the Trois Fontaines forest located in North-Eastern France (48°43′ N, 2°61′ W) is highly productive [17]. During the 2006–2010 period, the population growth rate was close to 0 at Chizé and 1.25 at Trois Fontaines and generation time was much longer at Chizé (about 7 years) than at Trois Fontaines (about 5 years) (Gaillard and Bonenfant, unpubl. data). As part of a long-term monitoring program, 9 to 12 days of capture were organized in each population between December and March, both in 2009–2010 and 2010–2011. Roe deer were captured using drive-netting [18] and blood samples were collected at the jugular vein. Samples were received at the laboratory within 40 hours after sampling and analyzed within 4 hours after reception. The sex, age (fawns *vs* older) and body mass (to the nearest 50g) of each captured roe deer were also recorded.

### Estimating Body Condition

We measured body condition using age class-specific body mass (MASS in kg) as well as six parameters designed to obtain detailed information on the metabolism of oxygen, proteins and carbohydrates.

We first estimated the aerobic capacity using red blood cell count (RBC in 10^12^/L) and hemoglobin concentration (HGB in g/dL). These indicators that reflect the presence of anemia have been previously used to assess body condition [7]. These measurements were issued from a complete blood count performed using an ABC Vet automaton (Horiba Medical, Montpellier, France). White and red blood cell counts (WBC, RBC) were measured by impedance technology considering parameters for bovine samples, since the size of blood cells is comparable between the two species [19]. Haemoglobin concentration was measured following cyan methemoglobin conversion at 550 nm, the most classical method in mammals [20]. An experienced laboratory technician performed a differential count of 100 WBCs and evaluated RBC, WBC and platelet morphology. Clotted and/or hemolytic serum samples were discarded.

The level of protein resources was estimated using albumin content (ALB in g/L), creatinin concentration (CREA in micromol/L), and urea (URE in mmol/L), which have all been previously used as indicators of body condition [21]. Albumin content is used to reflect the general level of available protein resources in serum [20]. In Ungulates, albumin level has been shown to be sensitive to population density, suggesting that this indicator may reflect the availability of resources [21]. Creatinin is produced during catabolism of muscular proteins, and reflects the intensity of muscular metabolism [20], while urea reflects the waste of the protein metabolism [20]. Classical biochemical methods were used to obtain these measurements. The total protein content of the serum was first measured using a refractometer [20]. Proteins were then separated into albumin, alpha-1, alpha-2, beta and gamma-globulins and quantified by electrophoresis, using an automatic agarose gel electrophoresis processor HYDRASYS (Sebia, Evry, France). Urea and creatinin analyses were performed on a Konelab 30i automaton (Fisher Thermo Scientific, Cergy-Pontoise, France) using Thermo scientific reagents.

To assess the level of carbohydrates, we used the level of fructosamine (FRU in micromol/L), which includes all carbohydrates combined to proteins and reflects glycemia during the two weeks preceding sampling [20]. Compared to glucose level, fructosamine concentration, being determined over several weeks before capture, has the advantage of being independent from capture stress. Fructosamin assays were performed on a Konelab 30i automaton (Fisher Thermo Scientific, Cergy-Pontoise, France) using ABX Pentrafructosamine reagents (Horiba, Montpellier, France).

### Measuring Immune Phenotype

As immunity has both innate and adaptive components that may have different determinants, and includes numerous cellular as well as humoral effectors, multiple measures reflecting these different aspects have to be analysed simultaneously [22]. Here, ten immune parameters were estimated using haematological parameters and specific assays. Concerning cellular immunity, neutrophils (NEUTRO in 10^12^/L) and monocytes (MONO in 10^12^/L) counts reflect acute and chronic inflammatory responses, respectively, and both may increase after infection. Eosinophils (EOSINO in 10^12^/L) are specifically induced by Th2 responses, but may be also present in other contexts like hypersensitivity [23]. Lymphocytes (LYMPHO in 10^12^/L) counts include both T and B cells, the latter being particularly involved in the production of antibodies and thus in adaptive responses [23]. These parameters were issued from the white blood cell count described above and from the distribution of white blood cells, which was determined by examination of wright-giemsa-stained blood smears. The neutrophils/lymphocyte ratio (N/L, dimensionless) was used as an integrative indicator of the inflammatory state [24], but this parameter is also known to be modified in stressful conditions [25].

Humoral aspects were first assessed using the levels of alpha-2 globulins (ALPHA2 in g/L) and haptoglobin (HAP in g/L). Alpha-2 globulins are a complex group of proteins that are produced during inflammatory response. Haptoglobin belongs to alpha-2 globulins, and is part of acute-phase proteins (APPs), a group of proteins which concentration changes following external or internal challenges such as trauma, inflammation or infection. In large herbivores, APP concentrations have been shown to increase during sarcoptic mange infestation [26]. Haptoglobin analyses were performed on a Konelab 30i automaton (Fisher Thermo Scientific, Cergy-Pontoise, France) using phase Haptoglobin assay (Tridelta Development LTD, County Kildare, Ireland) chromogenic kit.

Humoral innate immunity was also assessed by the levels of natural antibodies and complement. Natural antibodies are circulating antibodies that are present in the absence of any previous exposure to antigens. Their level in thus independent from the exposure of individuals to infection, but they are correlated to the ability to produce antibodies after a challenge [27] and have been used as a measure of immune allocation that correlates with fitness in large herbivores [28]. Their presence is revealed by hemagglutination (HA in –log2(dilution)), that measures the ability of samples to agglutinate exogenous cells. The complement is a group of proteins that acts through chain reactions and causes the lysis of exogenous cells in the presence of an antigen-antibody complex. They can thus be revealed by their ability to cause hemolysis (HL in –log2(dilution)) [27]. We used the hemagglutination-hemolysis protocol defined in [27], modified using chicken red blood cells as target cells.

Finally, we estimated the level of circulating antibodies, that are produced during adaptive response. The total amount of circulating antibodies has been used as a measurement of allocation in long-term immunity [6]. Here, we used the level of gamma-globulins (GAMMA in g/L) derived from the protein analysis described above as an estimator of total antibodies, since gamma-globulins are essentially constituted of circulating antibodies [20]. Overall, our main indicators of innate immunity were the neutrophil and monocyte counts, N/L ratio, alpha-2 globulin, haptoglobin and hemolysis, while hemagglutination and gamma-globulins were more reflecting adaptive response. Eosinophil and lymphocyte counts may reflect both aspects.

Haematological and biochemical assays were performed at the Biochemical and Endocrinological laboratory, VetAgro-Sup, France, while the hemagglutination-hemolysis assay was performed at the UMR 5558, Villeurbanne, France.

### Data Analysis

As body condition and immune phenotype were estimated using seven and ten parameters respectively, analyzing all possible relationships would lead to redundancies and high type I error. We thus analyzed the overall correlation pattern using a Principal Component Analysis (PCA). We then tested whether individuals from Chizé and Trois Fontaines differed in terms of body condition (hypothesis H1) and immune phenotype by comparing scores on the principal components (PCs), using linear models. Because the two populations differed in average score on PC1 (see results), we tested the contrast between populations for each variable using **t**-tests, or Wilcoxon tests when normality was not met.

To test our prediction that immunity correlates with body condition (H2, H3), we first defined a synthetic variable summarising body condition. This body condition index was the first principal component of a second PCA, including only body condition estimates for the same dataset, and showing a gradient similar to the one found in the complete PCA (not shown). We tested the relationship between each immune parameter and the body condition index using ANCOVAs to account for the between-population difference in immune phenotype. Variables were log-transformed or analyzed as presence/absence when normality was not met. All statistical procedures were performed using R software [29].

## Results

**Figure 1:**
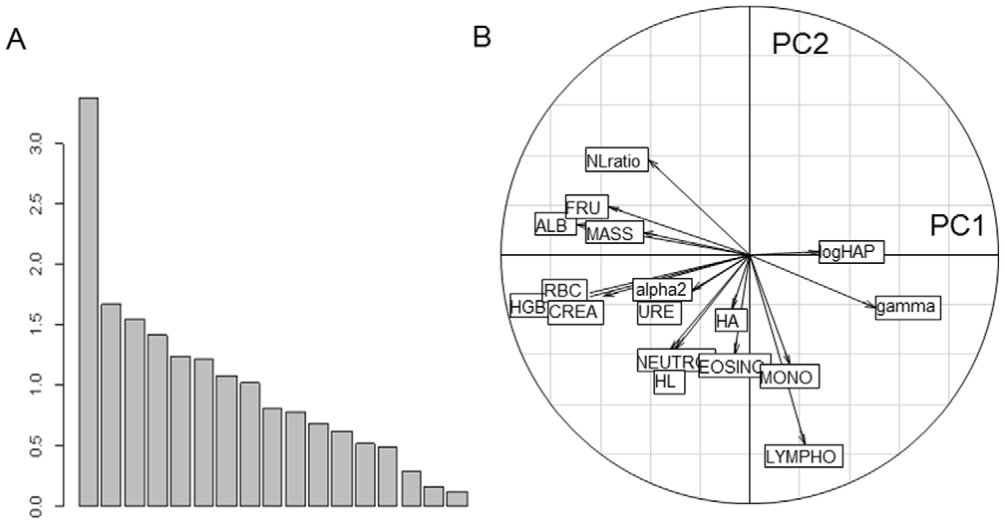
Body condition and immunocompetence. Principal Component Analysis for metabolic and immune parameters in two contrasting populations of roe deer in France. A: decomposition of variance among principal components; B: correlation circle showing the projection of all variables on principal components 1 x axis) and 2 (y-axis). See text for definition of variables.

**Figure 2:**
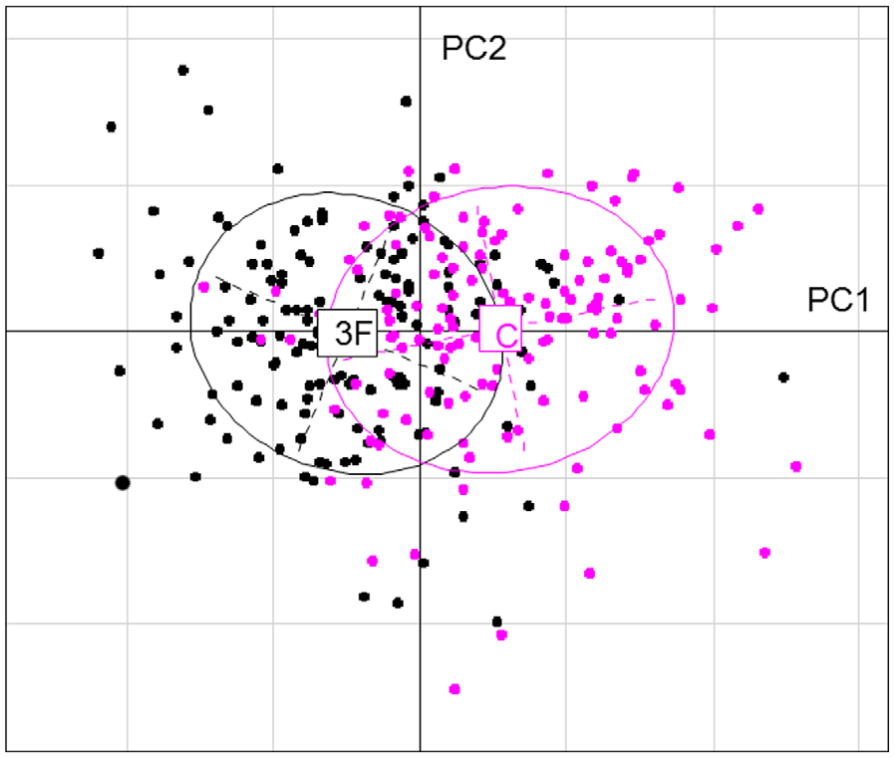
Contrast between populations. Projection of individuals from the two populations (C = Chizé, 3F = Trois Fontaines) on the first two principal component (displayed in the PC1-PC2 plane).

We analyzed 149 individuals from Trois Fontaines and 136 from Chizé (*n = *285). The proportion of males (0.47 vs. 0.41) did not differ between populations (Chi-square test, P = 0.325), while the proportion of fawns (0.33 vs. 0.18) was lower in Chizé (P = 0.007). The first principal component of PCA (PC1) captured 19.79% of the total inertia and was much more important than all following axes (Fig. 1), leading to retain only PC1 in the interpretation. All metabolic parameters correlated negatively with PC1. This first axis thus reflects a gradient of body condition, with individuals of high body condition having negative scores. As expected from hypothesis (H1) predicting a higher body condition in the population having access to more food resources, the ranking of roe deer along PC1 markedly differed between Chizé (mean score ± S.E = 1.10±0.13) and Trois Fontaines (−1.00±0.12, P<0.001, R^2^ = 0.32) with deer of highest body condition (low scores on PC1) belonging mostly to Trois Fontaines (Fig. 2). Accordingly, when taken separately, body mass and all metabolic parameters except URE were the highest at Trois Fontaines (all P<0.001). Because the age structure differed between Chizé and Trois Fontaines (*i.e*., larger proportion of fawns at Trois Fontaines), we tested if the difference between populations was confounded by an age effect. We found that the same patterns occurred (P<0.001) whether accounting for age or not.

Several immune parameters also varied along PC1. However, contrarily to expected from (H2), no general trend towards higher level of immunity at high body condition was observed. The strongest positive correlations occurred between PC1 scores and gamma-globulins (r = 0.50), haptoglobin (r = 0.27) and lymphocyte count (r = 0.22), while negative correlations were obtained between PC1 and N/L ratio (r = −0.41), hemolysis (r = −0.32) and neutrophil count (r = −0.30). Roe deer at Trois Fontaines had higher neutrophil counts (P<0.001) and hemolysis (P = 0.001). Again, these differences were confirmed after accounting for between-population difference in age structure, except for the N/L ratio that was significantly higher at Trois Fontaines only when the effect of age was accounted for (P = 0.012).

Body condition was therefore associated with immune phenotype, and this relationship remained after after accounting for between-population differences in immune parameters. Once the contrast in immune parameters between populations was accounted for, body condition index was positively correlated with N/L ratio (P = 0.006), eosinophil count (P = 0.032), haptoglobin (P = 0.005), alpha-2 globulins (P<0.001) and hemolysis (P = 0.038), and negatively associated with lymphocyte (P = 0.008) and monocyte counts (P = 0.009). These results are not supporting the hypothesis (H2) that high body condition should be associated with a high level of all immune parameters. Instead, high body condition was associated with high levels of N/L ratio, eosinophil count, alpha2-globulin, haptoglobin and hemolysis, and low levels of lymphocyte and monocyte counts. Whether these results support hypothesis (H3) is discussed below.

## Discussion

Measuring immunocompetence in wild populations is challenging, because of the limited access to animals, the impossibility to observe them for long periods and the stress induced by the capture. A limited set of immunological assays can be performed in these conditions. Moreover, these should be interpreted keeping in mind that the correlation between results of the assays and pathogen resistance is pathogen-dependent [22] and has generally not been assessed in wild species.

All indicators used to measure body condition co-varied positively, thus an overall metric of body condition can be defined based on classical measures of mass and metabolic parameters. As expected, body condition was much lower at Chizé than at Trois Fontaines. The low values of protein and carbohydrates reserves at Chizé suggest that resource limitation lowers the amount of nutrients available for the metabolism, as previously reported in white-tailed deer *Odocoileus virginianus* [21].

Immune parameters were, however, not consistently highest at Trois Fontaines or in roe deer with high body condition. The parameters associated with high body condition and/or Trois Fontaines population (neutrophil count, N/L ratio, alpha-2 globulin, haptoglobin and hemolysis) are clearly those expected to increase during a non-specific, acute and innate response. In contrast, individuals with low body condition or living at Chizé showed higher lymphocytes and monocytes counts (at low body condition) and high levels of gamma-globulins (at Chizé). This pattern is more difficult to interpret due to the low specificity of our indicators: because variations in T and B cells cannot be separated, lymphocyte count is not a straighforward indicator, and only gamma-globulins can be interpreted as a clear measure of adaptive immunity. This measure was higher at Chizé, but not in individuals having low body condition, within populations. Finally, at the level of population, although we compared only two sites, the contrast between Chizé and Trois Fontaines is consistent with species-level predictions that a slow pace of life, like observed at Chizé [30], should be associated with adaptive immunity [10]. At the individual level, due to the lack of clear relationship between body condition and adaptive response, our results partly support the prediction that, depending on their nutritional demands, individuals should allocate more resources either to innate, non-specific response or to adaptive (specifically, antibody-based) immunity [10].

Other determinants may also cause specific variations of the same parameters. The first possible confounding cause involves acute or chronic stress, which may modify leukocyte counts and N/L ratio [25]. Here, we measured creatine-kinase (CK) and aspartate transaminase (AST) as indirect indicators of acute stress in all captured roe deer. CK and AST reflect tissue injury and may increase dramatically after acute exercise, immobilization or cold exposure [20], [31]. On average, CK and AST were similar at Chizé and trois Fontaines and independent of body condition (not shown), suggesting that capture condition and tissue damage were of the same intensity in the two populations. We have no indication on how chronic stress may vary between populations. However the relationship between N/L ratio and body condition was maintained after considering the contrast between populations. Thus, the leukocyte distribution varied within populations among individuals experiencing similar capture conditions. We cannot reject the hypothesis that the immune phenotype observed in roe deer with high body condition, in particular high level of N/L ratio, might be due to stress in these individuals.

Other factors like sex, age and pregnancy may modulate immunity, thus these factors should be taken into account to explain the individual-level variation. Here, because the age structure differed between populations, we took the effect of age into account when comparing Chizé and Trois Fontaines. However, differences in age structure did not account for the differences we reported between populations.

Finally, individuals in low body condition might have experienced parasite infections that specifically entail adaptive immunity responses, thus parasite status has to be considered to interpret immune variation. Here, the parasite load has not been taken into account at the individual level. However, preliminary data and previous studies suggest that parasite burden is comparable between the two sites: in both populations, pathogens frequently encountered (prevalence >30%) include *Anaplasma phagocytophilum*, *Trichuris capreoli* and other gastro-intestinal strongyles [32], [33]. Less frequent pathogens (prevalence between 5 and 30%) include *Toxoplasma gondii* (Gotteland et al. submitted) and *Varestrongylus capreoli* [32]. A third group of parasites are occasionally found in both populations: *Coxiella burnetii*, *Nematodirus europaeus*, *Dictyocaulus noerneri*. Only *Capillaria sp*. has been found occasionally at Chizé and not at Trois Fontaines (Ferté et al., unpublished data). Other non-detected pathogens may be present at different levels in both populations. Here we have no clear indication of such situation since neutrophil and monocyte counts, which both increase after bacterial infection, do not show the same pattern.

Overall we found that the immune phenotype differed between populations and varied with body condition. Individuals living in a population with high resources and having high body condition showed high levels of parameters related to innate immunity, while the pattern was less clear for adaptive immunity. To further explore the variations in immune phenotype, monitoring individual trajectories while taking into account age, gender, pregnancy status, parasite pressure and direct measures of stress, could help identifying causes and consequences of the immune functioning.

## References

[1] Horrocks NPC, Matson KD, Tieleman BI (2011) Pathogen pressure puts immune defense into perspective. Integr Comp Pathol 51: 563–576 (doi: 10.1093/icb/icr011).. 10.1093/icb/icr011 21690107

[2] Lochmiller RL, Deerenberg C (2000) Trade-offs in evolutionary immunology: just what is the cost of immunity? Oikos 88: 87–98.

[3] Norris K, Evans MR (2000) Ecological immunology: life-history trade-offs and immune defense in birds. Behav Ecol 11: 19–26.

[4] Valderrabano J, Gomez-Rincon C, Uriarte J (2006) Effect of nutritional status and fat reserves on the periparturient immune response to *Haemonchus contortus* infection in sheep. Vet Parasitol 141: 122–131. 16737779 10.1016/j.vetpar.2006.04.029

[5] Martin LB, Navara KJ, Weil ZM, Nelson RJ (2007) Immunological memory is compromised by food restriction in deer mice *Peromyscus maniculatus* . Am J Physiol Regul Integr Comp Physiol 292: 316–320 (doi: 10.1152/ajpregu.00386.2006).. 10.1152/ajpregu.00386.2006 16902185

[6] Bourgeon S, Kauffmann M, Geiger S, Raclot T, Robin JP (2010) Relationships between metabolic status, corticosterone secretion and maintenance of innate and adaptive humoral immunities in fasted re-fed mallards. J Exp Biol 213: 3810–3818. 21037060 10.1242/jeb.045484

[7] Beldomenico PM, Telfer S, Gebert S, Lukomski L, Bennett M, et al (2008) Poor condition and infection: a vicious circle in natural populations. Proc R Soc Lond B 275: 1753–1759 (doi:10.1098/rspb.2008.0147).. 10.1098/rspb.2008.0147 PMC2453294 18448414

[8] Martin LBII, Weil ZM, Nelson RJ (2006) Refining approaches and diversifying directions in ecoimmunology. Integr Comp Biol 46: 1030–1039. 21672805 10.1093/icb/icl039

[9] Klasing KC (2004) The costs of immunity. Acta Zool Sin 50: 961–969.

[10] Lee KA (2006) Linking immune defenses and life history at the levels of the individual and the species. Integr Comp Biol 46: 1000–1015 (doi:10.1093/icb/icl049).. 21672803 10.1093/icb/icl049

[11] Leshchinsky TV, Klasing KC (2001) Divergence of the inflammatory response in two types of chickens. Dev Comp Immunol 25: 629–638. 11472784 10.1016/s0145-305x(01)00023-4

[12] Ksiazek A, Konarzewski M (2012) Effect of dietary restriction on immune Response of Laboratory mice divergently selected for basal metabolic rate. Physiol Biochem Zool 85: 51–61. 22237289 10.1086/663696

[13] Møller AP, Petrie M (2002) Condition dependence, multiple sexual signals, and immunocompetence in peacocks. Behav Ecol 13: 248–253.

[14] Masello JF, Choconi RG, Helmer M, Kremberg T, Lubjuhn T, et al (2009) Do leucocytes reflect condition in nestling burrowing parrots *Cyanoliseus patagonus* in the wild? Comp Biochem Physiol A- Mol & Integr Physiol 152: 176–181. 18854224 10.1016/j.cbpa.2008.09.018

[15] Krams I, Cirule D, Krama T, Vrublevska J (2011) Extremely low ambient temperature affects haematological parameters and body condition in wintering Great Tits (*Parus major*) J Ornithol. 152: 889–895 (doi: 10.1007/s10336-011-0672-7)..

[16] Andersen R, Gaillard JM, Linnell JD, Duncan P (2000) Factors affecting maternal care in an income breeder, the European roe deer. J Anim Ecol 69: 672–682.

[17] Gaillard JM, Delorme D, Boutin JM, Van Laere G, Boisaubert B, et al (1993) Roe deer survival patterns – a comparative analysis of contrasting populations. J Anim Ecol 62: 778–791.

[18] Pettorelli N, Gaillard JM, Mysterud A, Duncan P, Stenseth NC, et al (2006) Using a proxy of plant productivity (NDVI) to find key periods for animal performance: the case of roe deer. Oikos 112: 565–572 (doi: 10.1111/j.0030–1299.2006.14447.x)..

[19] Ursache O, Chevrier L, Blancou JM, Jaouen M (1980) Valeur des paramètres biochimiques et hématologiques chez le chevreuil (*Capreolus capreolus*). Rev Med Vet 131: 547–552.

[20] Stockham SL, Scott MA (2008) Fundamentals of veterinary clinical pathology, 2nd Edition. Blackwell Publishing, Ames (Iowa).

[21] Sams MG, Lochmiller RL, Qualls CW, Leslie DM (1998) Sensitivity of condition indices to changing density in a white-tailed deer population. J Wildl Dis 34: 110–125. 9476232 10.7589/0090-3558-34.1.110

[22] Adamo SA (2004) How should behavioural ecologists interpret measurements of immunity? Anim Behav 68: 1443–1449.

[23] Roitt I, Brostoff J, Male D (2001) Immunology. London: Mosby-Harcourt Publishers.

[24] De Jager CPC, van Wijk PTL, Mathoera RB, de Jongh-Leuvenink J, van der Poll T, et al (2010) Lymphocytopenia and neutrophil-lymphocyte count ratio predict bacteremia better than conventional infection markers in an emergency care unit. Critical Care 14: R192 (doi: 10.1186/cc9309).. 21034463 10.1186/cc9309 PMC3219299

[25] Davis AK, Maney DL, Maerz JC (2008) The use of leukocyte profiles to measure stress in vertebrates: a review for ecologists. Funct Ecol 22: 760–772.

[26] Rahman M, Lecchi C, Fraquell C, Sartorelli P, Ceciliani F (2010) Acute phase protein response in Alpine ibex with sarcoptic mange. Vet Parasitol 168: 293–298. 20036058 10.1016/j.vetpar.2009.12.001

[27] Matson KD, Robert E, Ricklefs RE, Klasing KC (2005) A hemolysis–hemagglutination assay for characterizing constitutive innate humoral immunity in wild and domestic birds. Develop Comp Immunol 29: 275–286 (doi:10.1016/j.dci.2004.07.006).. 10.1016/j.dci.2004.07.006 15572075

[28] Graham AL, Hayward AD, Watt KA, Pilkington JG, Pemberton JM, et al (2010) Fitness correlates of heritable variation in antibody responsiveness in a wild mammal. Science 330: 662–665. 21030656 10.1126/science.1194878

[29] R Development Core Team (2009) R: A language and environment for statistical computing. R Foundation for Statistical Computing, Vienna, Austria. ISBN 3–900051–07–0.

[30] Nilsen EB, Gaillard JM, Andersen R, Odden J, Delorme D, et al (2009) A slow life in hell or a fast life in heaven: demographic analyses of contrasting roe deer populations. J Anim Ecol 78: 585–594 (doi: 10.1111/j.1365–2656.2009.01523.x).. 19379139 10.1111/j.1365-2656.2009.01523.x

[31] Sanchez O, Arnau A, Pareja M, Poch E, Ramirez I, et al (2002) Acute stress-induced tissue injury in mice: differences between emotional and social stress. Cell Stress Chaperones 7: 36–46. 11892986 10.1379/1466-1268(2002)007<0036:asitii>2.0.co;2 PMC514800

[32] Segonds-Pichon A (2001) L’interaction hôte-parasite chez le chevreuil (*Capreolus capreolus*). Étude éco-épidémiologique de la relation entre les nématodes gastro-intestinaux et pulmonaires et la condition corporelle de l’hôte dans des populations contrastées. Unpublished PhD thesis, University Lyon 1.

[33] Body G, Ferté H, Gaillard JM, Delorme D, Klein F, et al (2011) Population density and phenotypic attributes influence the level of nematode parasitism in roe deer. Oecologia 167: 635–646. 21607671 10.1007/s00442-011-2018-9

